# REW-ISA: unveiling local functional blocks in epi-transcriptome profiling data via an RNA expression-weighted iterative signature algorithm

**DOI:** 10.1186/s12859-020-03787-w

**Published:** 2020-10-09

**Authors:** Lin Zhang, Shutao Chen, Jingyi Zhu, Jia Meng, Hui Liu

**Affiliations:** 1 Engineering Research Center of Intelligent Control for Underground Space, Ministry of Education, China University of Mining and Technology, Xuzhou, 221116 China; 2 School of Information and Control Engineering, China University of Mining and Technology, Xuzhou, 221116 China; 3 Department of Biological Sciences, AI University Research Center, Xi’an Jiaotong-Liverpool University, Suzhou, 215123 China

**Keywords:** m^6^A methylation, Iterative signature algorithm, Biclustering

## Abstract

**Background:**

Recent studies have shown that *N*^6^-methyladenosine (m^6^A) plays a critical role in numbers of biological processes and complex human diseases. However, the regulatory mechanisms of most methylation sites remain uncharted. Thus, in-depth study of the epi-transcriptomic patterns of m^6^A may provide insights into its complex functional and regulatory mechanisms.

**Results:**

Due to the high economic and time cost of wet experimental methods, revealing methylation patterns through computational models has become a more preferable way, and drawn more and more attention. Considering the theoretical basics and applications of conventional clustering methods, an RNA Expression Weighted Iterative Signature Algorithm (REW-ISA) is proposed to find potential local functional blocks (LFBs) based on MeRIP-Seq data, where sites are hyper-methylated or hypo-methylated simultaneously across the specific conditions. REW-ISA adopts RNA expression levels of each site as weights to make sites of lower expression level less significant. It starts from random sets of sites, then follows iterative search strategies by thresholds of rows and columns to find the LFBs in m^6^A methylation profile. Its application on MeRIP-Seq data of 69,446 methylation sites under 32 experimental conditions unveiled 6 LFBs, which achieve higher enrichment scores than ISA. Pathway analysis and enzyme specificity test showed that sites remained in LFBs are highly relevant to the m^6^A methyltransferase, such as METTL3, METTL14, WTAP and KIAA1429. Further detailed analyses for each LFB even showed that some LFBs are condition-specific, indicating that methylation profiles of some specific sites may be condition relevant.

**Conclusions:**

REW-ISA finds potential local functional patterns presented in m^6^A profiles, where sites are co-methylated under specific conditions.

## Background

*N*^6^-methyladenosine (m^6^A), which refers to the methylation of the adenosine bases at the nitrogen-6 position, is the most abundant post-transcriptional modification present in mRNAs and long non-coding RNAs [1]. It has been found to function in various pathways related to mRNA stability [2], DNA damage [3], differentiation [4], circadian clock [5], neurogenesis [6], immunity [7], anti-tumor activity [7], learning and memory [8], sex determination [9], heat shock response [10], etc. With the emergence of high-throughput sequencing technologies, Methylated Immunoprecipitation sequencing (MeRIP-Seq, or m^6^A-seq) [11, 12], researchers have been able to examine the dynamics and various functions of m^6^A in human, mouse, yeast, rice and other species [2, 5, 13–15].

The m^6^A methylation has been found to be governed or mediated by relevant enzymes, i.e., writers (METTL3/METTL14/WTAP complex [15, 16], KIAA1429 [17], VIRMA [18], RBM15 [19], ZC3H13 [20], etc.,), erasers (FTO [21], ALKBH5 [22], etc.,) as well as readers (YTH family [2, 10, 23–25], IGF2BP1-3 [26], eIF3 [27], etc.,). However, due to the complexity of life, the detailed regulatory circuit of RNA methylate remains uncharted, and it is believed to be more complex than enzyme induced mechanisms.

Till this day, several clustering methods have been proposed to identify co-methylation patterns in MeRIP-Seq data, trying to elucidate the functional mechanisms of m^6^A methylation. Liu et al*.* used four different clustering approaches, such as K-means, hierarchical clustering, Bayesian factor regression model as well as nonnegative matrix factorization to unveil the co-methylation patterns [15, 28]. To our knowledge, they revealed the linkage between the global co-methylation patterns embedded in epi-transcriptomic data for the first time. Cui et al*.* proposed MeTCluster to uncover the potential patterns of m^6^A methylation. It utilized a hierarchical graphical model to depict the reads counts, suggesting m^6^A functions could be location specific [29]. We have previously proposed an infinite beta binomial mixture model based on Dirichlet Process (DPBBM) to unveil the co-methylation patterns embedded in MeRIP-Seq data [30]. All the above-mentioned methods focused on clustering methylation sites under all conditions. Current studies have shown that on average 3–5 m^6^A RNA methylation sites position on each mRNA in human genome [31, 32]. It is conceivable that some specific sites may co-methylate under a subset of experimental conditions. Thus, clustering of sites over all conditions may miss biological meaningful information. On the one hand, sites sharing the same regulatory factor are more likely to co-methylate together; on the other hand, sites residing on the genes that belong to the same pathway may exhibit co-methylation patterns over subsets of experiments. Therefore, we aim to find some local functional blocks (LFBs), where sites are hyper-methylated or hypo-methylated simultaneously across the specific conditions in the same LFB, to unveil the local function patterns in m^6^A methylation profile.

Biclustering methods have been widely used to identify co-expressed genes under subsets of conditions in large scale microarray data [33–37]. Ihmels proposed an iterative signature algorithm (ISA) [34] to seek for biclusters, where subsets of co-regulated genes and conditions were selected by iterative searching procedure [35]. Murali et al*.* proposed Xmotifs algorithm, which takes a discretized gene expression matrix as input, to find co-expression patterns, where genes share the same expression level [36]. Prelić et al*.* proposed a Bimax method, which takes a binarized gene expression matrix as input, to find potential co-expression patterns [37]. The preprocess of discretization of input data results in serious information loss. When profiled by MeRIP-Seq technology, the quantification of RNA methylation level needs to be estimated from two complementary integer measurements indicating site reads count from input and IP samples. Conventionally, m^6^A methylation level is achieved by simple division operation, which calculates the percentage of site reads in IP sample over the total site reads of input and IP samples. However, it is not always accurate. Even if sites show the same percentage in value, their methylation levels maybe quite different due to their different RNA expression level. To be more specific, if the RNA expression level is very low, there may exist noise, which makes the percentage less confident. Therefore, we proposed herein an RNA Expression Weighted Iterative Signature Algorithm (REW-ISA), which adopted RNA expression level as weight to weaken the confidence sites, then followed an iterative search strategy through rows and columns to seek for LFBs. During the LFB searching strategy, each potential LFB is identified by column threshold (defined as *T*_*C*_) and row threshold (defined as *T*_*R*_). *T*_*C*_ and *T*_*R*_ are updated automatically according to Standard Deviation within Clusters (SDwC) and Average Similarity within Clusters (ASwC) metrics iteratively. SDwC indicates the closeness of each element in each LFB while ASwC indicates the correlation of each condition pair in each LFB.

REW-ISA was implemented on simulated data as well as real MeRIP-Seq induced m^6^A methylation level matrix to find potential LFBs. On simulated data, Score of Bi-Clustering (SoBC) metric was followed to evaluate the identification performance of LFBs. On real data, Gene Ontology (GO) analysis and enzyme specificity test were in the next conducted to validate the identified LFBs. As a result, REW-ISA can find LFBs that cover collaboratively hyper-methylated sites under specific conditions.

## Results

### Performance evaluation

In this study, we applied ISA as well as REW-ISA for simulated data biclustering for performance comparison. As is known, intersection over union (IoU) is a widely used evaluation metric in object detection, which is define as

1 $${\text{IoU}}\;{ = }\;\frac{{A_{o} }}{{A_{U} }}$$

where *A*_*O*_ represents the intersection between the obtained LFBs and ground truth, while *A*_*U*_ indicates the union of the obtained LFBs and ground truth. For example, suppose there are *s* LFBs embedded in simulated data, and *n* LFBs are obtained by clustering algorithm. In addition, let ${\varvec{G}} = \{ g_{1} , \ldots , g_{s} \}$ indicate whether there is uncovered LFB matching the *s* real LFBs respectively. At initialization, all elements in ***G*** are 0. So, we can calculate the IoU between each obtained LFB and the real LFBs. To be more specific, the IoU metric for the *i*-th obtained LFB and real one is achieved, and its maximum value is regarded as the final score of the *i*-th LFB, which is indicated by IoU_*id*_, representing the *i*-th uncovered LFB matches the *d*-th real LFB best. Thus, *g*_*d*_ = 1. For all the *n* identified LFBs, the average of IoU_*id*_ with *i* = 1, …, *n*, indicated as IoU_*mean*_ hereafter can be achieved. Since the number of obtained LFBs may differ from real, IoU_*mean*_ metric may not be sufficient for performance evaluation. Therefore, SoBC is defined to evaluate the agreement between REW-ISA obtained LFBs and ground truth.

2 $${\text{SoBC}} = \frac{r}{\max (s,n)}{\text{IoU}}_{mean}$$

where *r* indicates the number of ones in ***G***. Thus, *r* ≤ min(*s*, *n*). As SoBC approaches 1, the performance of biclustering is better.

In REW-ISA clustering procedure, *T*_*C*_ and *T*_*R*_ are key parameters for clustering stringency, and SDwC, ASwC scores are introduced to determine suitable *T*_*C*_ and *T*_*R*_. The mean and standard deviation of each LFB are combined in SDwC by (3).

3 $${\text{SDwC}} = \frac{\sqrt{\sum\limits_{k = 1}^{N}{\frac{1}{{m_{k} \cdot n_{k} }}\;\sum\limits_{i = 1}^{{m_{k} }} {\sum\limits_{j = 1}^{{n_{k} }} {\left( {w_{kij} p_{kij} - \overline{{\varvec{W}_{k} \varvec{P}_{k} }}} \right)^{2}}}}}}{N}$$

where *N* indicates the number of algorithm obtained LFBs, ***P***_***k***_ is the m^6^A methylation level of the *k*-th LFB, ***W***_***k***_ is the RNA expression weight for *k*-th LFB, *m*_*k*_ and *n*_*k*_ are the number of sites and conditions in the *k*-th LFB, *w*_*kij*_ is the RNA expression level of the *i*-th site under condition *j* in the *k*-th LFB, *p*_*kij*_ is the methylation level of the *i*-th site under condition *j* in the *k*-th LFB, $\overline{{{\varvec{W}}_{{\varvec{k}}} {\varvec{P}}_{{\varvec{k}}} }} = (1/m_{k} n_{k} )\sum\nolimits_{i = 1}^{{{\varvec{m}}_{{\varvec{k}}} }} {\sum\nolimits_{{{\varvec{j}} = 1}}^{{{\varvec{n}}_{{\varvec{k}}} }} {{\varvec{w}}_{{{\varvec{kij}}}} {\varvec{p}}_{{{\varvec{kij}}}} } }$. Thus, SDwC represents the standard deviation of methylation levels in each LFB.

ASwC is regarded as another concern for *T*_*C*_ and *T*_*R*_ selection. The pearson correlation between condition *a* and *b* in the *k*-th LFB is first calculated as *r*_*kab*_,

4 $$r_{kab} \; = \;\;\frac{{\sum\nolimits_{t = 1}^{{m_{k} }} {[(w_{kta} p_{kta} \; - \;\overline{{{\varvec{W}}_{{{\varvec{ka}}}} {\varvec{P}}_{{{\varvec{ka}}}} }} ){\kern 1pt} (w_{ktb} p_{ktb} \; - \;\overline{{{\varvec{W}}_{{{\varvec{kb}}}} {\varvec{P}}_{{{\varvec{kb}}}} }} )]} }}{{\sqrt {\sum\nolimits_{t = 1}^{{m_{k} }} {(w_{kta} p_{kta} \; - \;\overline{{{\varvec{W}}_{{{\varvec{ka}}}} {\varvec{P}}_{{{\varvec{ka}}}} }} )^{2} } } {\kern 1pt} \sqrt {\sum\nolimits_{t = 1}^{{m_{k} }} {(w_{ktb} p_{ktb} \; - \;\overline{{{\varvec{W}}_{{{\varvec{kb}}}} {\varvec{P}}_{{{\varvec{kb}}}} }} )^{2} } } }}$$

where ***W***_***ka***_ and ***W***_***kb***_ represent the RNA expression level under condition *a* and *b* in the *k*-th LFB, ***P***_***ka***_ and ***P***_***kb***_ represent the RNA methylation level under condition *a* and *b*, $\overline{{{\varvec{W}}_{{{\varvec{ka}}}} {\varvec{P}}_{{{\varvec{ka}}}} }} = (1/m_{k} )\sum\nolimits_{i = 1}^{{{\varvec{m}}_{{\varvec{k}}} }} {{\varvec{w}}_{{{\varvec{kia}}}} {\varvec{p}}_{{{\varvec{kia}}}} }$, $\overline{{{\varvec{W}}_{{{\varvec{kb}}}} {\varvec{P}}_{{{\varvec{kb}}}} }} = (1/m_{k} )\sum\nolimits_{i = 1}^{{{\varvec{m}}_{{\varvec{k}}} }} {{\varvec{w}}_{{{\varvec{kib}}}} {\varvec{p}}_{{{\varvec{kib}}}} }$. Then, ASwC is defined as

5 $${\text{ASwC}}\; = \;\frac{{\sqrt {\sum\nolimits_{k = 1}^{N} {\frac{2}{{n_{k} (n_{k} - 1)}}\;\sum\nolimits_{a = 1}^{{n_{k} }} {\sum\nolimits_{b = 1,b \ne a}^{{n_{k} }} {r_{kab} } } } } \;}}{N}$$

where *n*_*k*_ is the number of conditions in the *k*-th LFB. Thus, ASwC indicates how the involved sites co-methylate between conditions in each LFB.

Our original intention is to better reveal the biological functional mechanisms of the co-methylation modules based on transcriptome data, larger SDwC and smaller ASwC metrics are preferred to get larger LFBs with more implicit information.

### Simulated data

**Fig. 1:**
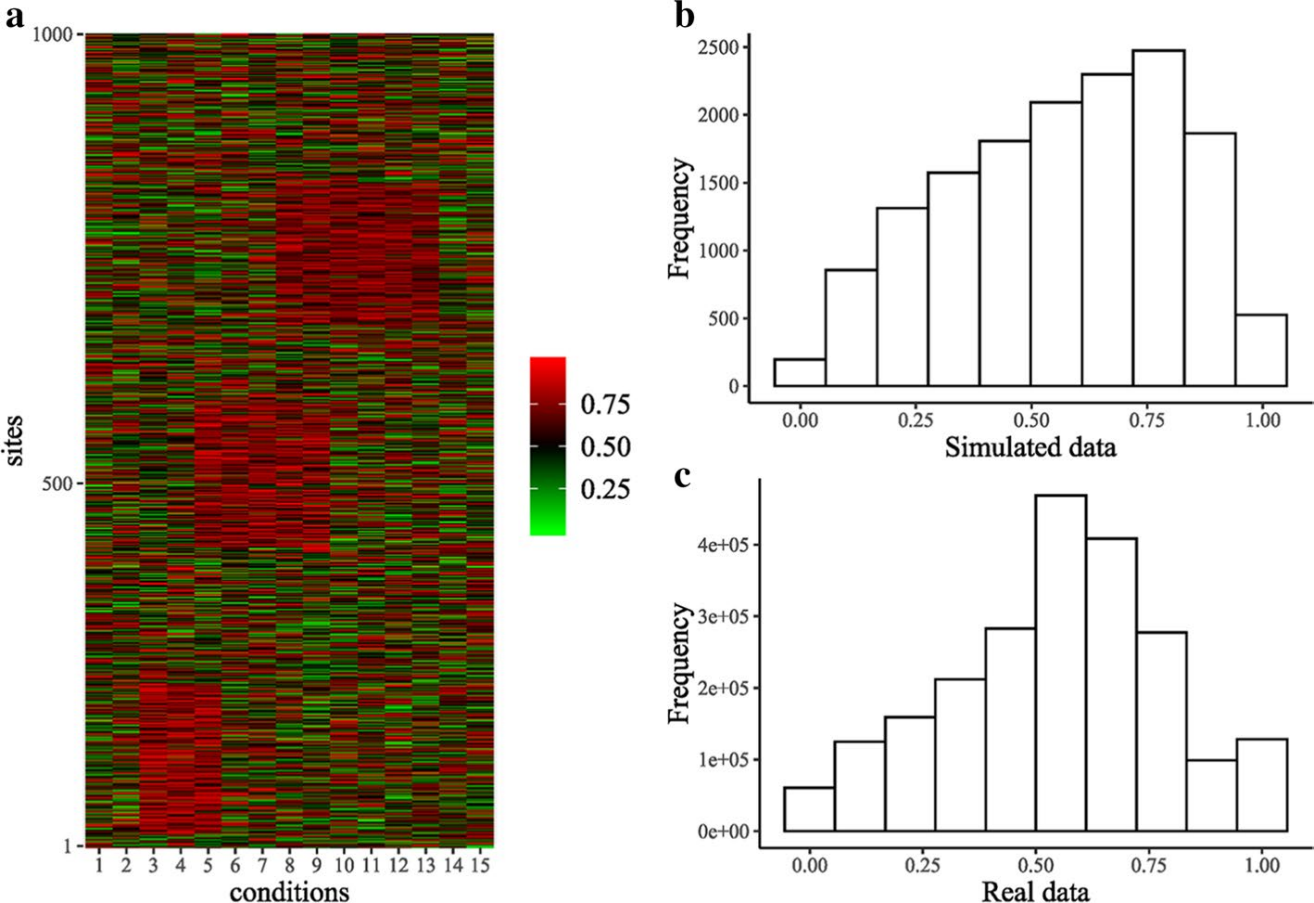
Comparison of statistical characteristics between simulated data and real data. **a** Heatmap of simulated data. Rows are corresponding to m^6^A sites while columns represent conditions. **b** Histogram of simulated data. **c** Histogram of real data

For performance evaluation, a simulated RNA methylation dataset of size 1000 × 15 was generated from a mixture of 4 beta-binomial distributions, corresponding to three biclustering blocks and the background (Fig. 1a). The overall distribution characteristics of the simulated data were set similar to that of the real MeRIP-Seq data (Fig. 1b, c) to mimic real scenarios. The matched methylation expression data, which is used as the “weight” in the proposed algorithm, was directly calculated from the simulated RNA methylation dataset as previously described.

As is known, *T*_*C*_ and *T*_*R*_ are defined for subset selection along columns and rows in conventional ISA. They are also defined in REW-ISA, and play a decisive role in LFB stringency. Since the methylation level matrix is *p* ≫ *n*, it is intuitive that the conditions should be selected more carefully, thus *T*_*C*_ asks for more cautious exercise, and detailed explanations are given in method section.

**Fig. 2:**
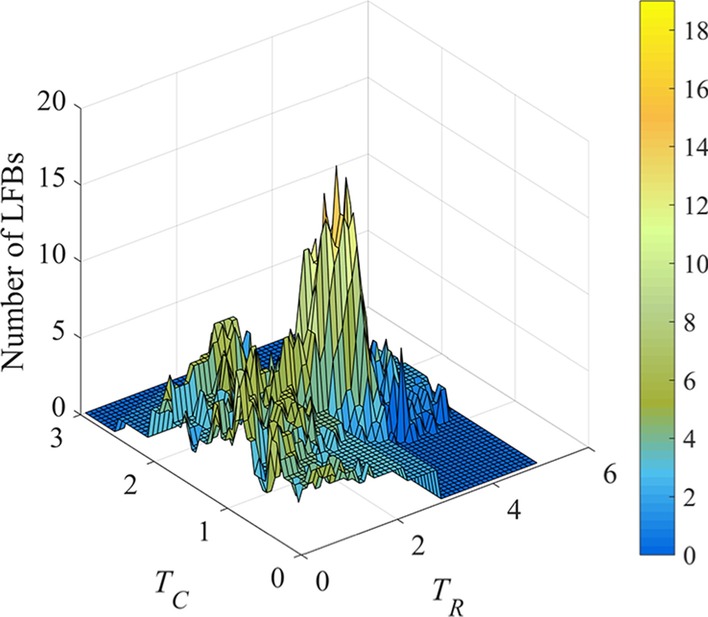
The number of LFBs identified by REW-ISA with *T*_*R*_ set from 0.1 to 5 at step size of 0.1, *T*_*C*_ set from 0.05 to 3 at step size of 0.05. The color scale indicates the number of LFBs obtained

In REW-ISA, a grid search method was followed for parameter optimization. The range of *T*_*R*_ is 0.1–5 with step size 0.1, and the range of *T*_*C*_ is 0.05–3 with step size 0.05. Their upper bounds are recommended a large value, then adapted automatically in following procedures. For each {*T*_*R*_, *T*_*C*_} setting, 40 experiments were conducted to ensure the robustness. After repeated experiments, the mode of LFB number was adopted as final number of LFBs for each threshold pair, as shown in Fig. 2.

According to Fig. 2, the range of *T*_*R*_ and *T*_*C*_ can be shrunk. To be more specific, the thresholds that obtain the largest number of LFBs were first reached, then filter out all the combinations with larger *T*_*R*_ or *T*_*C*_. This is because, in the abandoned combinations, REW-ISA can also get the same number of LFBs with smaller *T*_*R*_ and *T*_*C*_. However, smaller *T*_*R*_ and *T*_*C*_ remain more rows and columns in each LFB, which may unveil more useful biological information. Thus, the range of *T*_*R*_ becomes 0.1–2.5, while the range of *T*_*C*_ becomes 0.05–1.15.

We can see from Fig. 2 that when *T*_*R*_ and *T*_*C*_ are close to their lower limits, only a few LFBs can be achieved with very large scale, which cannot uncover implicit information for functions. Thus, REW-ISA raises the lower bound of *T*_*R*_ and *T*_*C*_ appropriately. Suppose the matrix of LFB number obtained under different threshold setting is ${\varvec{L}} \in {\mathbb{R}}^{rn \times cn}$ with *T*_*R*_ and *T*_*C*_ adjusted, where *rn* represents the number of *T*_*R*_s and *cn* represents the number of *T*_*C*_s considered. Min–max normalization of ***L*** is performed to obtain ${\varvec{L}}^{{{\varvec{norm}}}} \in {\mathbb{R}}^{rn \times cn}$, and then the variance of each row and column in ***L***^***norm***^ is calculated. The variance of the *i*-th row in ***L***^***norm***^ is *vr*_*i*_ (1 ≤ *i* ≤ *rn*), and the variance of the *j*-th column is *vc*_*j*_ (1 ≤ *j* ≤ *cn*).

6 $$vr_{i} = \frac{{\sum\nolimits_{j = 1}^{cn} {\left(l_{ij}^{norm} -\frac{1}{cn}\sum\nolimits_{j = 1}^{cn} l_{ij}^{norm} \right)^{2}} }}{cn}$$

7 $$vc_{j} = \frac{{\sum\nolimits_{i = 1}^{rn} {\left(l_{ij}^{norm} - \frac{1}{rn}\sum\nolimits_{i = 1}^{rn} l_{ij}^{norm} \right)^{2} } }}{rn}$$

Furthermore, the mean values of elements in ***vr*** and ***vc*** are calculated as *vr*_*mean*_ and *vc*_*mean*_, respectively. We set $i^{\prime} = \min \{ i:vr_{i} \ge vr_{mean} \}$ and $j^{\prime} = \min \{ j:vc_{j} \ge vc_{mean} \}$, and then drop out the first *i′ *− 1 values of the *T*_*R*_ and the first *j′ *− 1 values of the *T*_*C*_ from consideration. Thus, the range of *T*_*R*_ further becomes 0.3–2.5, while the range of *T*_*C*_ becomes 0.1–1.15.

After shrinking the range of *T*_*R*_ and *T*_*C*_, the matrix of LFB numbers under each threshold pair setting is updated to be $\user2{L^{\prime}} \in {\mathbb{R}}^{rn^{\prime} \times cn^{\prime}}$, where $rn^{\prime} = rn - i^{\prime} + 1$ and $cn^{\prime} = cn - j^{\prime} + 1$. Then, within the selected threshold range of *T*_*R*_ and *T*_*C*_, a sliding window of size *η*_*r*_ × *η*_*c*_ is used to help find more stable selections of *T*_*R*_ and *T*_*C*_. The value of *η*_*r*_ and *η*_*c*_ are selected by Eqs. (8) and (9),

8 $$\eta_{r} = 2 \times \left\lceil {\frac{0.1}{{step_{r} }}} \right\rceil + 1$$

9 $$\eta_{c} = 2 \times \left\lceil {\frac{0.1}{{step_{c} }}} \right\rceil + 1$$

where *step*_*r*_ represents the variable step size of *T*_*R*_, *step*_*c*_ represents that of *T*_*C*_, and $\left\lceil \cdot \right\rceil$ is round up to integer operation. The sliding window is obvious to cover odd number of rows and columns, which makes the thresholds value of interest locate in the center of the sliding window. Specifically, for the element *l′*_*ij*_ locating in the *i*-th (1 ≤ *i* ≤ *rn′*) row and the *j*-th (1 ≤ *j* ≤ *cn′*) column of ***L′***, the mode of the values covered by sliding window is calculated, then compared to the center value *l′*_*ij*_. If they are equal, the threshold setting is maintained for further consideration, and *ls′*_*ij*_ = 1 is recorded in the matrix ${\varvec{LS}} \in {\mathbb{R}}^{rn^{\prime} \times cn^{\prime}}$. Otherwise, *ls′*_*ij*_ = 0. It is worth noting that when sliding the elements on the boundary of ***L′***, we only select the effective elements in the sliding window to test the stability.

Through ***L′*** and the stable score matrix ***LS***, the threshold pairs with stable LFB number can be screened out. Let ***S*** = ***L′*** × ***LS***, ${\varvec{S}} \in {\mathbb{R}}^{rn^{\prime} \times cn^{\prime}}$, the threshold pairs corresponding to non-zero elements in ***S*** are stable threshold combinations. After filtering out 0 in ***S***, the number of obtained LFBs in ***S ***are counted to provide frequency *f*_*ln*_, where *ln* represents the number of LFBs, and the result is shown in Table 1.

**Table 1:** Statistics of the number of LFBs obtained in the simulated data

*ln*	2	**3**	4	5	6	7	9	14
*f*_*ln*_	0.133	**0.445**	0.244	0.093	0.057	0.017	0.003	0.008

The bold value in the table is the maximum value of the frequency

*ln* represents the number of LFBs obtained; and *f*_*ln*_ represents the frequency at which the number of LFBs is *ln*

According to the statistics of the number of stable LFBs, that is, Table 1, we find that the number of LFBs with the highest frequency is 3. Therefore, we can reasonably conclude that there is a total of 3 LFBs in the simulated data.

**Fig. 3:**
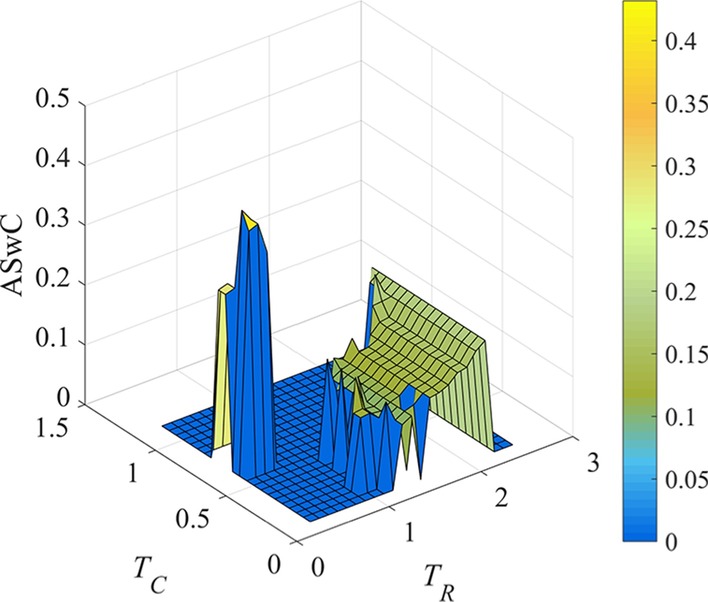
ASwC metrics of LFBs identified by REW-ISA, the number of LFBs obtained by each threshold pair is 3. The color scale indicates the value of ASwC

Furthermore, the threshold pairs in ***LS*** are filtered according to the number of obtained LFBs is 3. At the same time, we update the ASwC calculated under effective settings of *T*_*R*_ and *T*_*C*_. The ASwC value obtained by effective threshold pair is shown in Fig. 3.

Because ASwC is used to measure the similarity between columns in LFBs, the smaller ASwC is, the more information is contained in each LFB. Since smaller ASwC can help retrieve more biological meaningful information, we calculate the mean of ASwC achieved under effective threshold pairs, and remain the threshold pairs with which ASwC scores are less than the mean score to further shrink the range of *T*_*R*_ and *T*_*C*_.

**Fig. 4:**
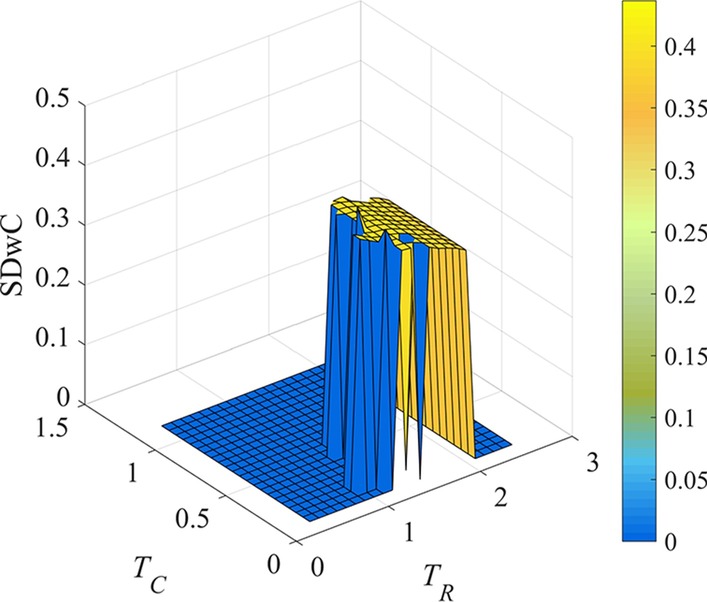
SDwC metrics of LFBs identified by REW-ISA, the values of *T*_*R*_ and *T*_*C*_ are their respective shrinking threshold ranges. The color scale indicates the value of SDwC

Within the narrowed *T*_*R*_ and *T*_*C*_ range, the SDwC values of each threshold pair are further compared, and the result is shown in Fig. 4.

As shown in Fig. 4, the optimal value of *T*_*R*_ and *T*_*C*_, where SDwC gets its maximum indicating the loose and information abundance of each LFB, are 1.2 and 0.35 respectively. Since smaller threshold may find the larger LFB, REW-ISA chooses smaller *T*_*R*_ and *T*_*C*_ when the maximum value of SDwC is achieved under multiple pairs of *T*_*R*_ and *T*_*C*_.

In a word, it is suggested that the upper bound initialization of *T*_*R*_ and *T*_*C*_ be set to larger values, and REW-ISA can automatically shrink the range. Besides, the step size of *T*_*R*_ and *T*_*C*_ during grid search has no effect on final result. The REW-ISA thresholds optimization algorithm is in the following.

**Table:** 

**Algorithm 1:** Thresholds optimization process of REW-ISA
**Input:** Methylation level matrix ***P***, weight matrix ***W*** and initialize the range of *T*_*R*_ and *T*_*C*_
**Output:** The optimal *T*_*R*_ and *T*_*C*_, and the number of LFBs determined by the above *T*_*R*_ and *T*_*C*_
**Step1:** Run REW-ISA within the initial threshold range, obtain the threshold pair that generates the most LFBs, and then shrink the range of *T*_*R*_ and *T*_*C*_
**Step2:** Within the threshold range after contraction, the LFB number matrix ***L′***, stable score matrix ***LS***, stable LFB number matrix ***S***, compactness SDwC and ASwC are calculated
**Step3:** Count the frequency of the number of LFBs in the matrix ***S***
**Step4:** According to the maximum frequency, select the corresponding optimal number of LFBs
**Step5**: The ASwC value corresponding to each threshold pair is calculated, and the thresholds ranges are further reduced
**Step6**: Select the optimal *T*_*R*_ and *T*_*C*_ according to the maximum SDwC within the selected thresholds range
**Return** The optimal *T*_*R*_ and *T*_*C*_, and the number of LFBs determined by the optimal *T*_*R*_ and *T*_*C*_

**Fig. 5:**
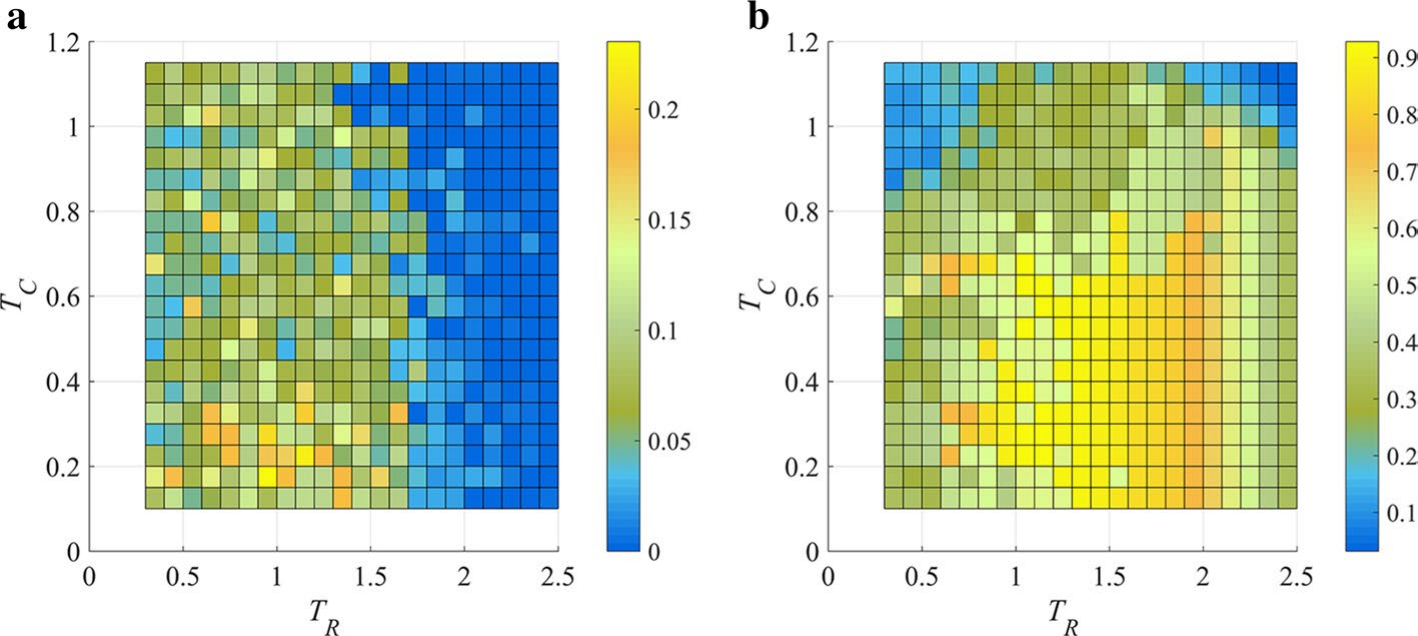
Heatmap of SoBC with *T*_*C*_ varying from 0.05 to 1.5, and *T*_*R*_ varying from 0.1 to 2.5. **a** Heatmap of SoBC obtained by ISA. **b** Heatmap of SoBC obtained by REW-ISA. The color scale indicates SoBC score

To validate the automatic parameter selection procedure of *T*_*R*_ and *T*_*C*_, we investigated the SoBC of ISA and REW-ISA identified LFBs with varying *T*_*C*_s (0.1 to 1.15) and *T*_*R*_s (0.3 to 2.5), as shown in Fig. 5.

As shown Fig. 5b, in REW-ISA, the optimal value of *T*_*R*_ and *T*_*C*_ locate between 1.2–1.4 and 0.1–0.65 respectively on simulated data. This is consistent with the beforementioned parameter selection procedure given in Algorithm 1. Besides, we also found that the SoBC metric of REW-ISA can reach around 0.9, while that of ISA is around 0.2, implying that the LFBs uncovered by REW-ISA is more effective than ISA.

### Real data

A total of 69,446 human m^6^A sites identified by six base-resolution mi-CLIP and m^6^A-CLIP experiments were obtained by WHISTLE project [38–42]. However, mi-CLIP and m^6^A-CLIP only report the positioning of m^6^A sites, but do not provide the methylation level of each site. The information of methylation level still comes from MeRIP-Seq data. To be more specific, 32 samples in 10 publicly human m^6^A MeRIP-Seq data sets were collected [2, 5, 11, 17, 43–47], and most of them can be retrieved from MeTDBV2.0 database [48]. The detailed description of data was given in additional file (see Additional file 1: Table S1).

As is known, MeRIP-Seq data profiles the m^6^A epi-transcriptome by input and IP data. Thus, we first followed [42, 49] to quantify methylation level of each site. The biological replicates of the same cell line from the same experiment were merged, and the methylation level of the combined samples is essentially the average of all the biological replicates. All the sequencing data were downloaded in SRA format from Gene Expression Omnibus, and the reads were aligned to human reference genome hg19 with Tophat2 (with default settings as *read-mismatches* = 2, *read-gap-length* = 2, *read-edit-dist* = 2, *min-anchor* = 8, *min-intron-length* = 50 and *max-intron-length* = 500,000) for Fragments Per Kilobase of transcript per Million (FPKM) statistics [50].

The methylation level was then quantified by calculating the ratio of fold enrichment of reads in IP sample over the total of IP and input samples. To be more specific, let *t*_*ij*_ representing FPKM of the *i*-th site in IP sample under the *j*-th condition, and *h*_*ij*_ representing FPKM of the *i*-th site in input sample under the *j*-th condition. Let ***P*** indicate the methylation level matrix, the methylation level of the *i*-th site under the *j*-th condition *p*_*ij*_ can be calculated following (10).

10 $$p_{ij} = \frac{{t_{ij} { + }\alpha }}{{t_{ij} + h_{ij} { + }2\alpha }}$$

where *α* is a very small value, aiming to avoid *NaN* where FPKM of both IP and input samples are zeros, and *p*_*ij*_ resides in (0,1).

We also constructed the weight matrix ***W*** corresponding to ***P***,

11 $$w_{ij} = \log (t_{ij} + h_{ij} + 1)$$

where 1 was added to ensure *w*_*ij*_ ≥ 0. With the employment of ***W***, the less confident sites with lower expression level are weakened for further biclustering analysis.

Then, REW-ISA is conducted based on ***P*** and ***W***. Within the range of *T*_*R*_ being 0.1–5 with step size 0.1, and *T*_*C*_ being 0.05–3 with step size 0.05, *T*_*R*_ and *T*_*C*_ are optimized through grid search method. The experiments were repeated 10 times for each parameter setting.

**Fig. 6:**
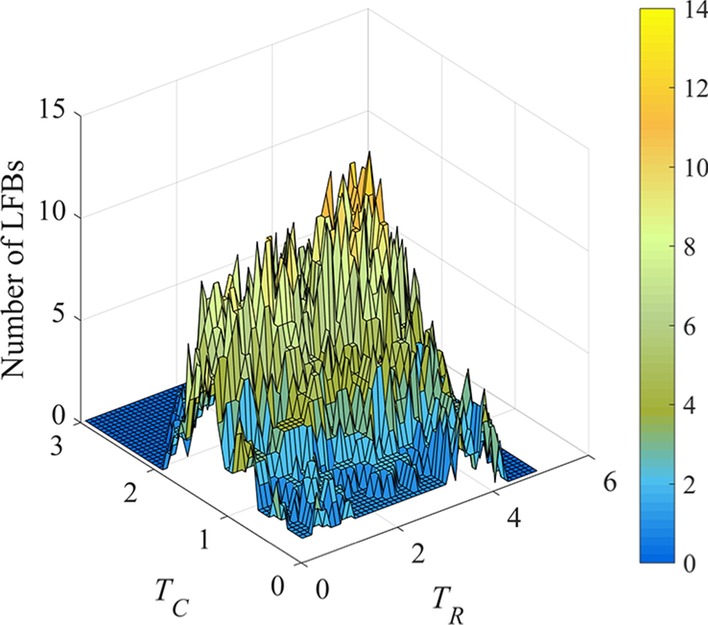
The number of LFBs identified by REW-ISA with *T*_*R*_ ranges in 0.1–5 with step size 0.1, *T*_*C*_ ranges in 0.05–3 with step size 0.05. The color scale indicates the number of LFBs obtained

As shown in Fig. 6, maximum number of LFBs is 14. The upper bounds of *T*_*R*_ and *T*_*C*_ are 3.5 and 1.35 respectively. Furthermore, the variances of each row and column in the LFB number matrix are calculated according to (6) and (7), and then the mean values of row variances and column variances are calculated respectively. The first elements which is larger than the above mean values are selected from the obtained row and column variance vectors, and the corresponding *T*_*R*_ and *T*_*C*_ are the new lower bounds of *T*_*R*_ and *T*_*C*_. Based on the above process, the lower bounds of *T*_*R*_ and *T*_*C*_ are set to 1.3 and 0.6. The statistics of the number of LFBs obtained under different *T*_*R*_ and *T*_*C*_ are shown in Table 2. Thus, the number of LFBs is preferred to be 6.

**Table 2:** Statistics of the number of LFBs obtained in the real data

*ln*	2	3	4	5	**6**	7	8	9	10	11	12
*f*_*ln*_	0.06	0.12	0.06	0.16	**0.22**	0.08	0.08	0.07	0.09	0.05	0.01

The bold value in the table is the maximum value of the frequency

*ln* represents the number of LFBs obtained; and *f*_*ln*_ represents the frequency at which the number of LFBs is *ln*

**Fig. 7:**
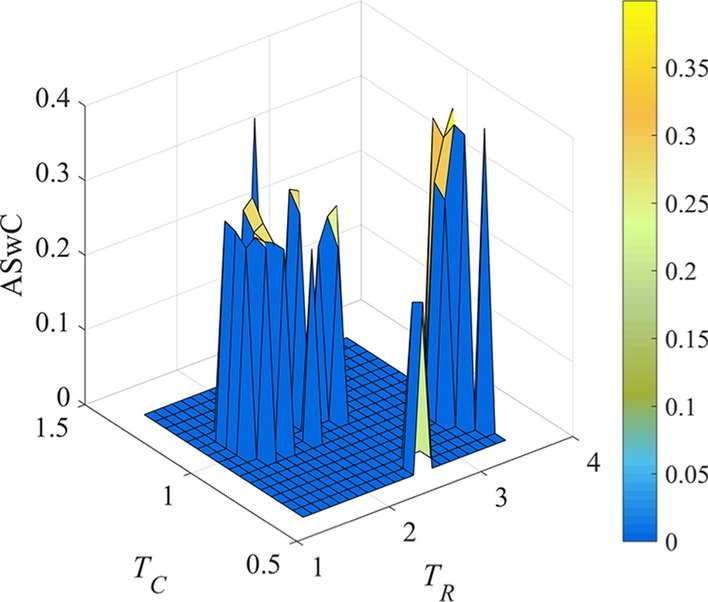
ASwC metrics of LFBs identified by REW-ISA, the number of LFBs obtained by the threshold pairs is 6. The color scale indicates the value of ASwC

Based on the threshold pairs that achieve 6 LFBs, ASwC scores are presented in Fig. 7.

**Fig. 8:**
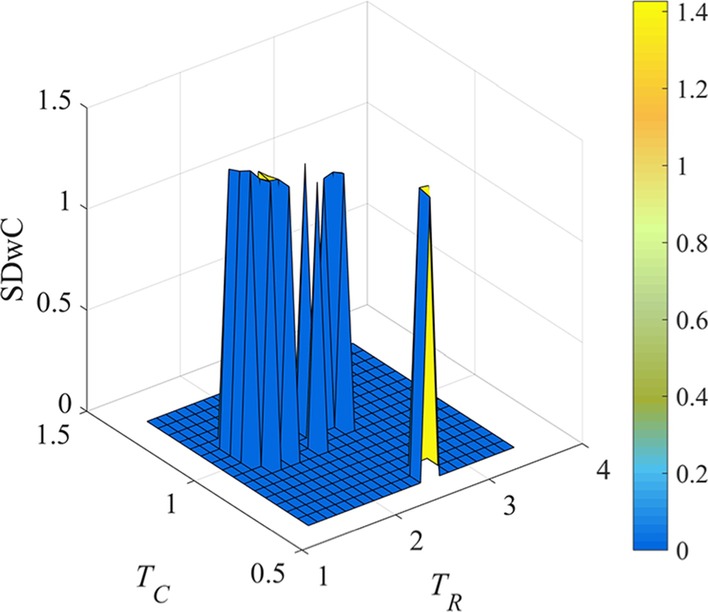
SDwC metrics of LFBs identified by REW-ISA, the values of *T*_*R*_ and *T*_*C*_ are their respective shrinking threshold ranges. The color scale indicates the value of SDwC

Then, the mean of ASwC scores is calculated, and the threshold pairs that get greater ASwC than the mean is further filtered. For the remained threshold pairs, their corresponding SDwC values are calculated, as shown in Fig. 8.

Based on Fig. 8, *T*_*R*_ and *T*_*C*_ are selected to be 1.6 and 1 as optimal, where the largest SDwC appears.

To further explore the biological relevance of the reported six LFBs, we first annotated the Entrez Gene ID and Gene Symbol of genes corresponding to each site in each LFB, then conducted pathway and GO enrichment analysis. Six KEGG pathways known to be regulated by RNA methylation [3, 11, 21] were selected to validate whether a pathway is significantly enriched in a specific LFB using Fisher’s exact test. The output *p*-value shows the significance of association between the obtained LFBs and the biological pathways with multiple hypothesis corrections.

We could see from Table 3 that obtained LFB1, LFB2 and LFB3 are significantly enriched in fatty acid metabolism. Fatty acids are a substance of the aliphatic group, and the efficacy and function of fatty acids are mainly supplemented for human absorption. Also, studies have found that fatty acids play important roles in regulating metabolism, growth and development and cell differentiation. LFB2 and LFB3 are also enriched in p53 pathway, which consists of a network responding to a variety of intrinsic and extrinsic stress signals that impact upon cellular homeostatic mechanisms, disrupting DNA replication, chromosome segregation and cell division, etc. [51, 52]. As is known, Gamma and UV irradiation could also result in DNA damage [53]. LFB6 is shown to be significantly enriched in both Fatty Acid Metabolism and UV response Down pathways, implying that LFB6 is composed of methylation sites that are relevant to life development process from fatty acid metabolism affected by UV stimulation. For LFB5, it is not enriched to any of the six KEGG pathways, indicating that LFB5 may have other implicit biological significance, and further analysis is carried out in the next.

**Table 3:** Pathway analysis of REW-ISA obtained LFBs

ID	Number of sites	Enrichment statistics	KEGG pathways					
			Apoptosis	DNA repair	Fatty acid metabolism	p53 pathway	UV response down	UV response Up
LFB1	4780	OR	0.9939	0.5541	0.2156	1.5781	2.2294	1.4360
		*p*-value	1.0000	0.1891	**0.0113**	0.0655	**0.0032**	0.1887
		FDR	1.0000	0.3219	0.0665	0.1573	0.0437	0.3219
LFB2	4834	OR	1.0623	0.5375	0.2092	1.7047	2.3044	1.2789
		*p*-value	0.7558	0.1470	**0.0114**	**0.0251**	**0.0019**	0.4122
		FDR	0.8245	0.2940	0.0665	0.1006	0.0437	0.5712
LFB3	4899	OR	0.9241	0.8954	0.3044	1.8062	2.2083	1.4464
		*p*-value	1.0000	0.8738	**0.0336**	**0.0129**	**0.0041**	0.1967
		FDR	1.0000	0.9253	0.1209	0.0665	0.0437	0.3219
LFB4	5440	OR	0.7568	0.8152	0.4260	1.4756	1.6945	1.3718
		*p*-value	0.4760	0.6583	0.0531	0.0775	**0.0453**	0.2341
		FDR	0.6119	0.7406	0.1469	0.1744	0.1469	0.3664
LFB5	5713	OR	0.7447	0.8027	0.4624	1.5126	1.4158	1.2249
		*p*-value	0.4125	0.5729	0.0629	0.0526	0.1856	0.4756
		FDR	0.5712	0.6874	0.1573	0.1469	0.3219	0.6119
LFB6	4714	OR	0.7329	0.7878	0.2251	1.5574	2.1795	1.3776
		*p*-value	0.5174	0.6185	**0.0162**	0.0834	**0.0049**	0.3094
		FDR	0.6423	0.7183	0.0729	0.1766	0.0437	0.4641

The values with *p*-value less than 0.05 are shown in bold in the table

OR stands for odds ratio; *p*-value is evaluated by Fisher’s exact test; and the FDR is calculated with BH method

The GO enrichment analysis was then conducted by clusterProfiler Bioconductor package [54] for each obtained LFB, with *p*-value cutoff set as 0.05 and *q*-value cutoff 0.2. For all the GO terms enriched by genes in each LFB, the negative log transform of *p*-value was employed as their enrichment scores.

12 $$s_{i} = - \log (p_{i} )$$

where *p*_*i*_ is the *p*-value of the *i*-th GO term.

**Fig. 9:**
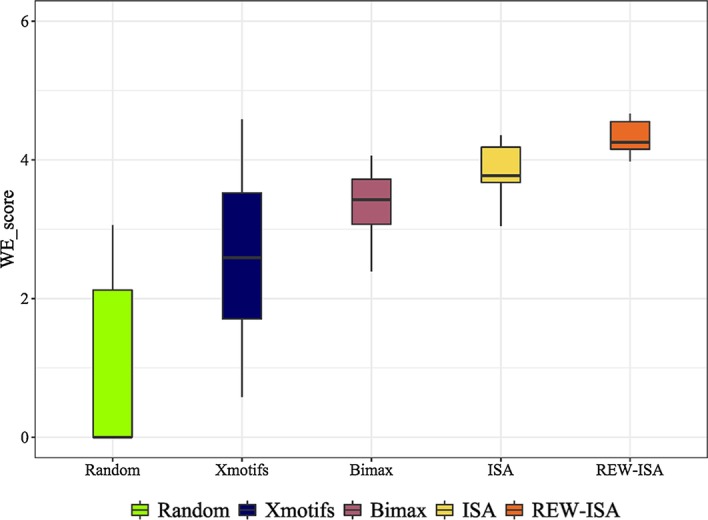
WE_score of LFBs obtained by random gene groups and four biclustering algorithms

In fact, GO terms with more enriched genes may not show higher enrichment scores. As to LFBs, the proportion of genes that involved in LFB is also an important factor. It is conceivable that we adhere a weight to better describe the contribution of each GO term. The weight of the *i*-th GO term is defined as *m*_*i*_/*M*, where *m*_*i*_ is the number of genes of the *i*-th GO term enriched in this LFB and *M* is the total number of genes in this LFB. Therefore, WE_score is defined as (13).

13 $${\text{WE}}\_{\text{score}} = \frac{{s_{1} m_{1} /M + s_{2} m_{2} /M + \cdots + {\kern 1pt} s_{l} m_{l} /M}}{{m_{1} /M + m_{2} /M + \cdots + {\kern 1pt} m_{l} /M + m_{non} \;{/}\;M}}$$

where *l* is the number of GO terms that enriched in each LFB, *m*_*non*_ is the number of genes covered by LFB but not enriched by any GO term. The higher the WE_score is, the more biologically significant the LFBs are [55]. Three state of the art algorithms, Xmotifs [36], Bimax [37], ISA [35] were conducted for biclustering on real data in comparison to REW-ISA. Besides, subsets with different number of sites and conditions were selected randomly as LFBs from real data. WE_score for all the obtained LFBs by all the algorithms are given in Fig. 9.

It can be seen from Fig. 9 that the REW-ISA algorithm is effective, and the result is consistent with many related research results [37, 56]. The average WE_score of LFBs inferred by REW-ISA is 13.2% higher than that of ISA, which implied more biological significance of REW-ISA. Besides, LFBs identified by the four algorithms achieve significantly higher WE_score than random one, which also indicates the biological significance.

We further examined whether the identified LFBs show enzyme’s substrate specificity. Since LFB covers hyper-methylated sites and conditions, the sites and conditions involved in each LFB are more likely to be the target sites of m^6^A methyltransferases. Therefore, we investigated the association between each LFB and four m^6^A methyltransferases, including METTL3, METTL14, WTAP as well as KIAA1429. For this purpose, 12,643 METTL3 targeted sites, 7689 METTL14 targeted sites, 13,124 WTAP-targeted sites and 399 KIAA1429 targeted RNA methylation sites were first identified by TREW [48], as shown in Table 4.

**Table 4:** Number of m^6^A methyltransferase target sites in each LFB

ID	Methyltransferase component			
	METTL3	METTL14	WTAP	KIAA1429
LFB1	3605	3916	4303	267
LFB2	3728	3989	4342	287
LFB3	3888	4207	4490	296
LFB4	4432	4569	4991	345
LFB5	4712	4635	5255	344
LFB6	3728	3989	4342	287

Then, the association between the sites in each LFB and m^6^A methyltransferases target sites was further evaluated by Fisher’s exact test. The reported *p*-value indicates the significance of association between sites and methyltransferase target sites. As shown in Table 5, all the four m^6^A methyltransferases targeted sites in the six obtained LFBs are significantly enriched (FDR < 0.05), which means the LFBs obtained by REW-ISA were indeed the collaboratively hyper-methylated sites under specific conditions.

**Table 5:** Enzyme specificity analysis of REW-ISA obtained LFBs

ID	Number of sites	Enrichment statistics	Methyltransferase component			
			METTL3	METTL14	WTAP	KIAA1429
LFB1	4780	OR	2.5617	14.7698	9.8933	2.4626
		*p*-value	5.87E−184	0	0	2.71E−33
		FDR	7.83E−184	0	0	2.71E−33
LFB2	4834	OR	2.8308	15.4638	9.6866	2.6610
		*p*-value	1.28E−221	0	0	1.19E−40
		FDR	1.92E−221	0	0	1.25E−40
LFB3	4899	OR	3.2549	20.2677	12.1375	2.7250
		*p*-value	7.73E−278	0	0	1.80E−43
		FDR	1.24E−277	0	0	2.05E−43
LFB4	5440	OR	3.7806	17.8603	12.4793	2.9442
		*p*-value	0	0	0	4.22E−56
		FDR	0	0	0	5.33E−56
LFB5	5713	OR	4.0817	14.6430	12.9915	2.7716
		*p*-value	0	0	0	1.09E−50
		FDR	0	0	0	1.30E−50
LFB6	4714	OR	2.6572	18.1924	11.2583	2.7263
		*p*-value	3.71E−194	0	0	2.82E−42
		FDR	5.23E−194	0	0	3.07E−42

**Fig. 10:**
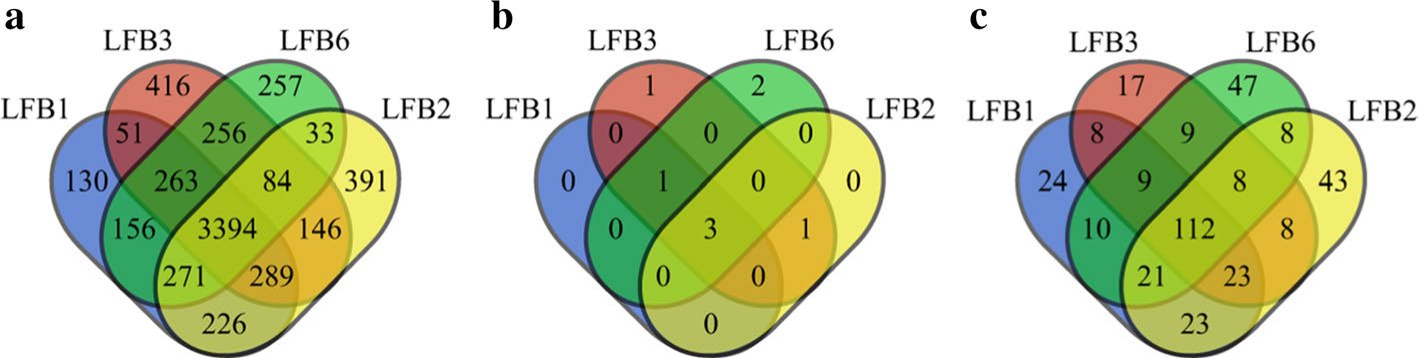
Venn diagrams for obtained LFBs. **a** Venn diagrams of sites in LFB1, LFB2, LFB3 and LFB6. **b** Venn diagrams of conditions in LFB1, LFB2, LFB3 and LFB6. **c** Venn diagrams of functional annotations of genes that selected sites in LFB1, LFB2, LFB3 and LFB6 reside on

In LFB1, LFB2, LFB3 and LFB6, Venn diagrams of the sites, conditions and functional annotations of genes that selected sites involved in each LFB reside on were shown in Fig. 10. As shown in Fig. 10a, it was obvious that this four LFBs contain 12,971 identical methylation sites. From the perspective of conditions, the conditions involved in LFB1 were all covered by LFB3 and LFB6, while LFB3 and LFB6 contain two conditions that were not contained by LFB1, respectively, as shown in Fig. 10b. It is also worth mentioning that for LFB2, all the conditions included in it are from human liver hepatocellular cells (HepG2) cell lines, indicating some LFBs may be condition specific. Although the conditions contained in LFB1 and LFB3 are very similar, they still contain over one hundred unshared functional annotations, which may be due to site differences between them, as shown in Fig. 10c.

**Fig. 11:**
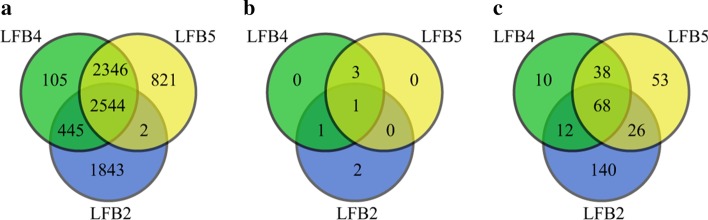
Venn diagrams for obtained LFBs. **a** Venn diagrams of sites in LFB2, LFB4 and LFB5. **b** Venn diagrams of conditions in LFB2, LFB4 and LFB5. **c** Venn diagrams of functional annotations of genes that selected sites in LFB2, LFB4 and LFB5 reside on

Since LFB2 was found enriched in three KEGG pathways previously, and might be condition specific, LFB2 was compared with LFB4 and LFB5 for further study, and the Venn diagram is shown in Fig. 11.

It can be seen from Fig. 11 that although LFB2, LFB4 and LFB5 share 2544 sites, they share only one condition, which leads to 203 functional annotations that are not shared at all, indicating that the three LFBs may play different roles in m^6^A methylation.

**Fig. 12:**
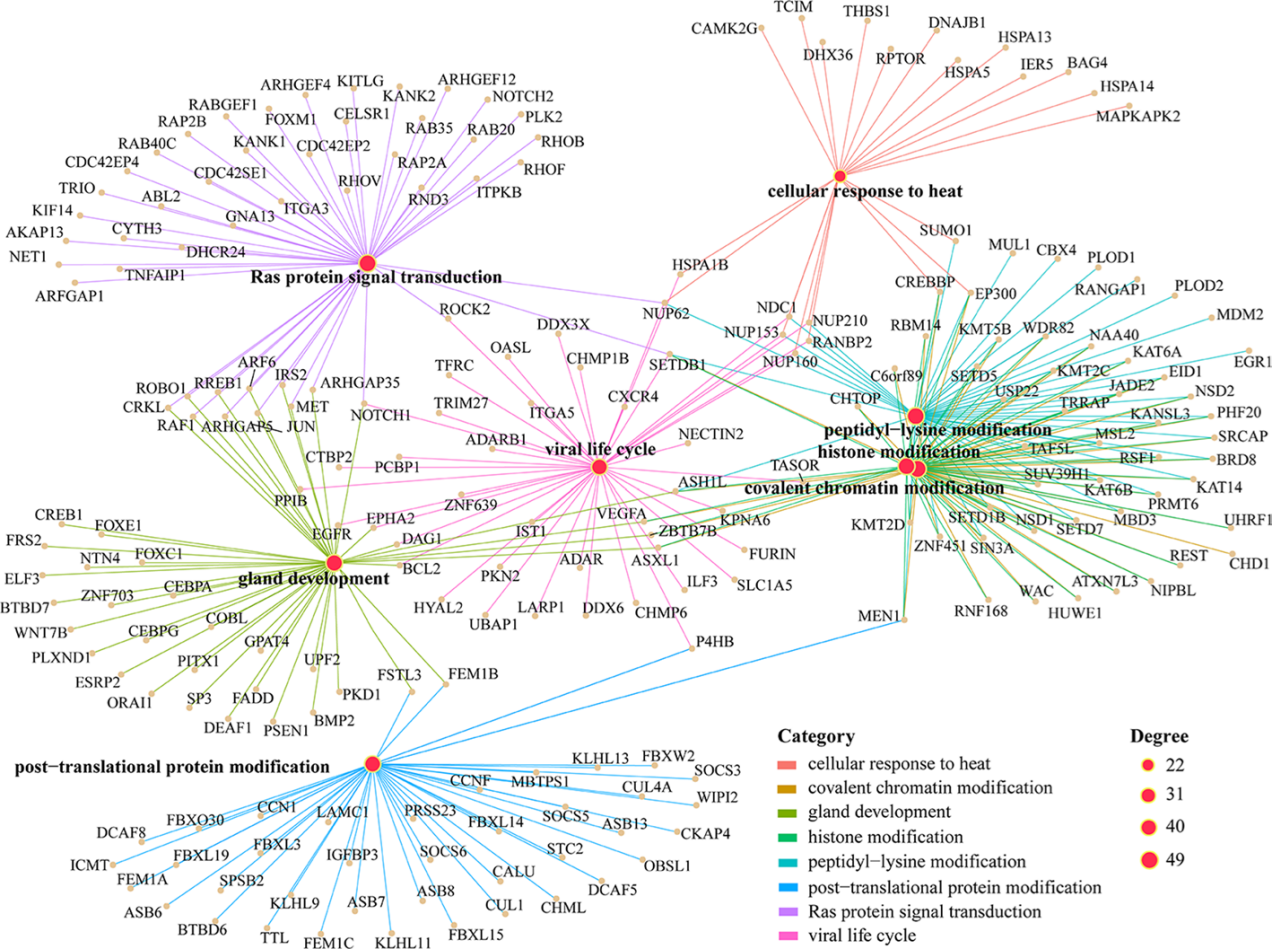
The functional relationship diagram obtained from the analysis of the genes related to the LFB2 using KEGG pathway. Degree represents the number of genes enriched by KEGG pathway

We further investigated the functions of LFB2 in detail, as shown in Fig. 12.

Some genes that sites in LFB2 reside on are found to be involved in m^6^A-related pathways, such as Ras protein signal transduction [57], macromolecule methylation [58], peptidyl-lysine modification [59], histone modification [60] and covalent chromatin modification [61], implying LFB2 may further help elaborate the functional mechanisms of m^6^A methylation. Besides, some pathways, such as response to heat [10, 62], are found to be significantly enriched in LFB2, which is also consistent with previous analysis that LFB2 covers conditions with HepG2 cells that exposed to ultraviolet radiation, heat shock, hepatocyte growth factor (HGF; also known as scatter factor (SF)), and interferon-*γ*.

**Fig. 13:**
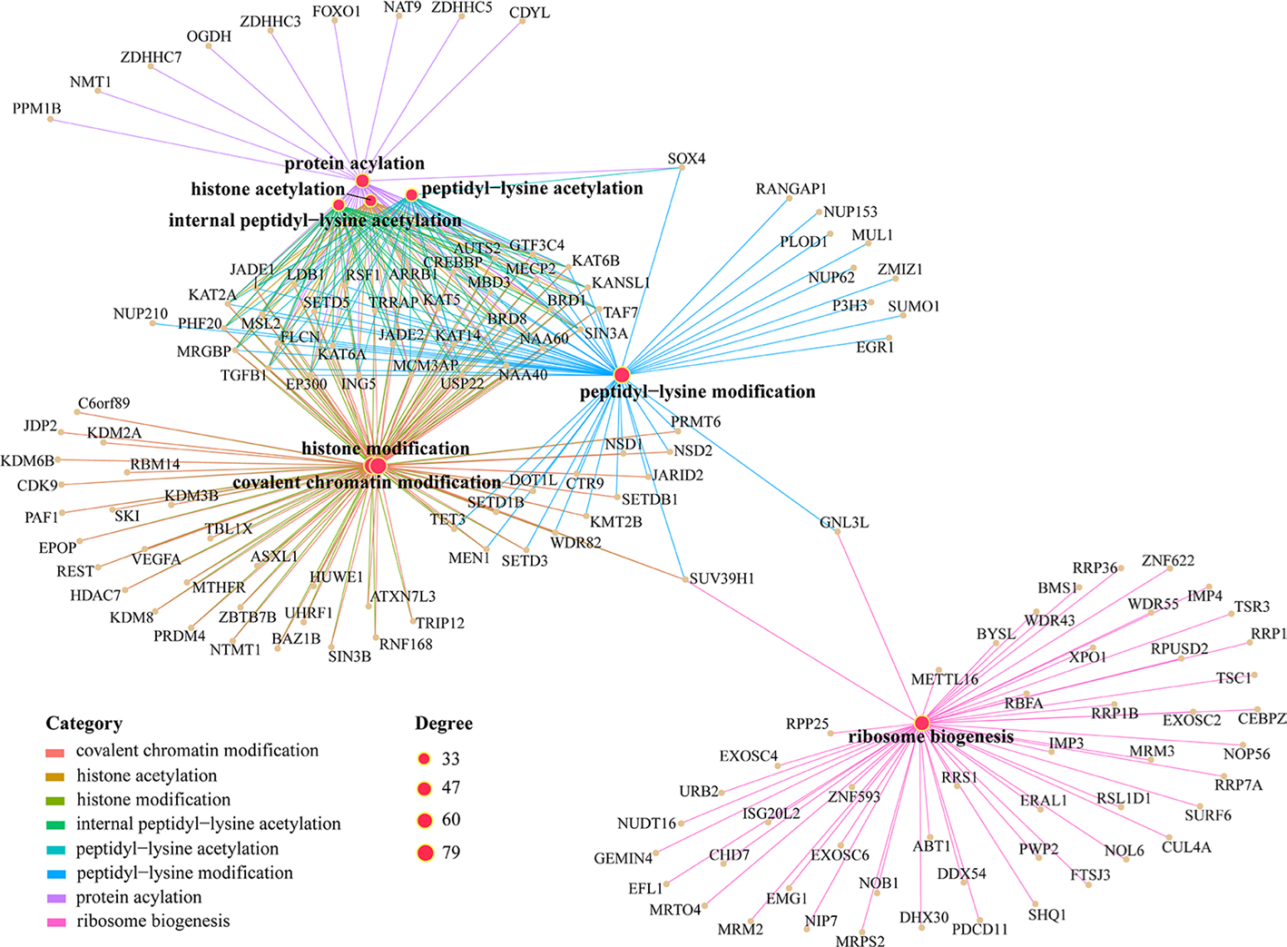
The functional relationship diagram obtained from the analysis of the genes related to the LFB5 using KEGG pathway. Degree represents the number of genes enriched by KEGG pathway

Since LFB5 is not enriched in any of the six KEGG pathways in the previous analysis, the functionality of LFB5 is similarly examined in further detail, and the result is shown in Fig. 13.

We can see that LFB5 is mainly enriched in functional annotations related to histone and lysine modification, in which the modification form is mainly acetylation modification. M^6^A modification in RNA has been found to be determined by histone modification [63]. Therefore, the genes contained in LFB5 may help uncover the relationship between histone and lysine modification and m^6^A methylation.

## Discussion

More and more studies have shown that m^6^A RNA methylation plays an extremely important role in a variety of biological processes. Moreover, the functions of m^6^A methylation have been revealed by more and more researchers. Through the study of m^6^A methylation, we could understand the pathogenesis of the disease at post-transcriptional level, which would help us build a more comprehensive understanding of life process such as disease mechanisms. However, unveiling the functional m^6^A methylation sites through biological experiments is time-consuming and expensive, so it is very necessary to develop some effective computational algorithms to predict potential functional m^6^A sites. In this paper, we developed an RNA expression weighted ISA method, REW-ISA, to uncover the potential local methylation patterns across subsets of condition. REW-ISA approached 6 LFBs based on MeRIP-Seq data from 10 cell lines under 32 different conditions. Further GO analysis and some specificity tests show that REW-ISA obtained LFBs can find hyper-methylated local functional patterns, which are highly relevant with conditions.

REW-ISA could achieve reliable biclustering patterns because of its adoption of RNA expression level. For the m^6^A methylation level matrix, the level was drawn based on the ratio between IP and input samples, and there are no additional supplements for RNA expression level. To be more specific, the methylation levels of sites of high expression level should be more confident than those of low expression level since the reads count statistics in low expression sites may come from noise, which makes them unconfident. By incorporation of RNA expression level, sites with very low abundance of reads count will be assigned very low weight, thus, excluded for consideration of biclustering. Of course, REW-ISA still has some deficiencies that needs to be improved in the future. REW-ISA seek for LFBs of hyper-methylated sites under subsets of conditions based on methylation level, which is achieved by simple division operation. However, it may lead to information loss during the division operation. The information carried by both input and IP samples should be more than the methylation level and RNA expression level. In the future, we will develop new computational model to overcome these limitations.

## Conclusions

With comparison with conventional ISA method, we believe that our test suggests REW-ISA as a simple but effective tool for local functional pattern recognition tasks. Through the experiments, we also showed that REW-ISA is also feasible for real-world applications with similar issues as local pattern analysis problem in m^6^A methylation profiles.

## Methods

**Fig. 14:**
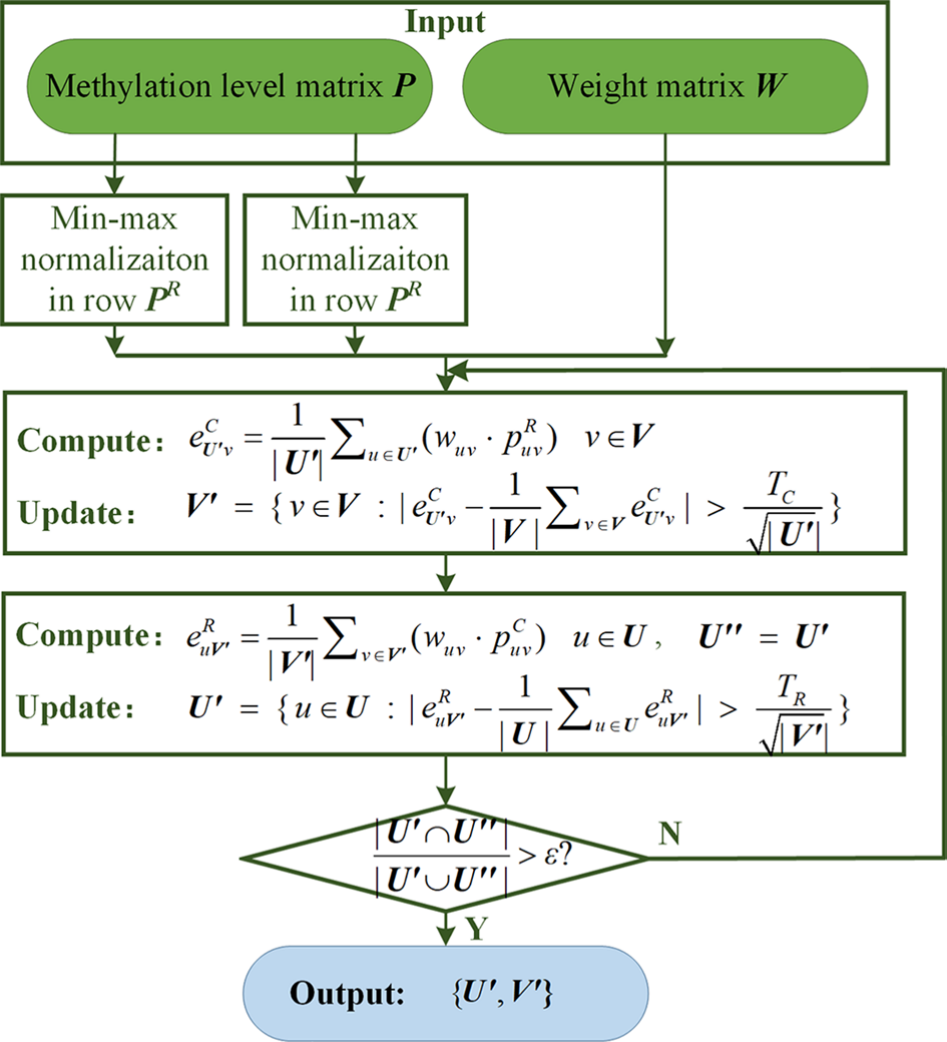
The flowchart of REW-ISA consists of two steps: The first step prepares the methylation level matrix ***P*** and weight matrix ***W***; the second step iteratively updates subsets for LFBs. The iterative update refers to the iterative selection along columns and rows

In conventional ISA method, rows and columns of data are standardized first, and subsets of rows and columns are updated iteratively according to their own thresholds. However, in REW-ISA, we propose to import weights to enhance the confidence of methylation level estimation, so the min–max normalization was employed instead of z-score normalization. The methylation level matrix ${\varvec{P}} \in {\mathbb{R}}^{p \times n}$ turns into ***P***^***R***^ after row min–max normalization, and turns into ***P***^***C***^ after column min–max normalization. The flowchart of REW-ISA is shown in Fig. 14. In general, REW-ISA consists of two steps. The first step aims to form the methylation level and weight matrix for all sites under all conditions. The second part conducts iteratively selection of subsets of rows and columns for LFBs. With the employment of ${\varvec{W}} \in {\mathbb{R}}^{p \times n}$, the contribution of sites showing similar methylation level may be distinguishable due to their different expression level.

For subset selection of columns, the updated subsets are achieved following (14).

14 $$\left\{ \begin{gathered} e_{{\user2{U^{\prime}}{\kern 1pt} v}}^{C} \; = \;\frac{1}{{|\user2{U^{\prime}}{|}}}\sum\nolimits_{{u\; \in \;\user2{U^{\prime}}}} {\left(w_{{u{\kern 1pt} v}} \; \cdot \;p_{{u{\kern 1pt} v}}^{R} \right)} {\kern 1pt} {\kern 1pt} {\kern 1pt} {\kern 1pt} {\kern 1pt} {\kern 1pt} {\kern 1pt} {\kern 1pt} {\kern 1pt} {\kern 1pt} {\kern 1pt} {\kern 1pt} {\kern 1pt} {\kern 1pt} {\kern 1pt} {\kern 1pt} {\kern 1pt} {\kern 1pt} {\kern 1pt} {\kern 1pt} {\kern 1pt} {\kern 1pt} {\kern 1pt} {\kern 1pt} {\kern 1pt} {\kern 1pt} {\kern 1pt} {\kern 1pt} {\kern 1pt} {\kern 1pt} {\kern 1pt} {\kern 1pt} {\kern 1pt} {\kern 1pt} {\kern 1pt} {\kern 1pt} {\kern 1pt} {\kern 1pt} {\kern 1pt} {\kern 1pt} v\; \in \;{\varvec{V}} \hfill \\ \user2{V^{\prime}}\;\; = \;\;\left\{ v\; \in {\varvec{V}}\;\;:\;\;|e_{{\user2{U^{\prime}}{\kern 1pt} v}}^{C} - \frac{1}{{|{\varvec{V}}|}}\sum\nolimits_{{v\; \in \;{\varvec{V}}}} {e_{{\user2{U^{\prime}}{\kern 1pt} v}}^{C} } |\;\; > \;\;\frac{{T_{C} }}{{\sqrt {|\user2{U^{\prime}}} {|}}}\right\} \hfill \\ \end{gathered} \right.$$

where ***V*** is the column set of ***P***, $p_{{u{\kern 1pt} v}}^{R}$ refers to the *u*-th site under *v*-th condition in ***P***^***R***^, *w*_*uv*_ is the RNA expression level of the *u*-th site under *v*-th condition. In Eq. (14), $e_{{U^{\prime}{\kern 1pt} v}}^{C}$ is calculated based on ***P***^***C***^ and ***W*** for column subset selection, but only the conditions involved in ***U′ ***are considered. Higher *T*_*C*_ setting will result in less conditions in LFB.

Then, the subsets of rows are updated following (15).

15 $$\left\{ \begin{gathered} e_{{u{\kern 1pt} \user2{V^{\prime}}}}^{R} \; = \;\frac{1}{{|\user2{V^{\prime}}{|}}}\sum\nolimits_{{v\; \in \;\user2{V^{\prime}}}} {(w_{{u{\kern 1pt} v}} \; \cdot \;p_{{u{\kern 1pt} v}}^{C} )} {\kern 1pt} {\kern 1pt} {\kern 1pt} {\kern 1pt} {\kern 1pt} {\kern 1pt} {\kern 1pt} {\kern 1pt} {\kern 1pt} {\kern 1pt} {\kern 1pt} {\kern 1pt} {\kern 1pt} {\kern 1pt} {\kern 1pt} {\kern 1pt} {\kern 1pt} {\kern 1pt} {\kern 1pt} {\kern 1pt} {\kern 1pt} {\kern 1pt} {\kern 1pt} {\kern 1pt} {\kern 1pt} {\kern 1pt} {\kern 1pt} {\kern 1pt} {\kern 1pt} {\kern 1pt} {\kern 1pt} {\kern 1pt} {\kern 1pt} {\kern 1pt} {\kern 1pt} {\kern 1pt} {\kern 1pt} {\kern 1pt} {\kern 1pt} {\kern 1pt} u \in {\varvec{U}} \hfill \\ \user2{U^{\prime}}\;\; = \;\;\left\{ {u\; \in \;{\varvec{U}}\;\;:\;\;|e_{{u{\kern 1pt} \user2{V^{\prime}}}}^{R} - \frac{1}{{|{\varvec{U}}|}}\sum\nolimits_{{u\; \in \;{\varvec{U}}}} {e_{{u{\kern 1pt} \user2{V^{\prime}}}}^{R} } |\;\; > \;\;\frac{{T_{R} }}{{\sqrt {|\user2{V^{\prime}}} {|}}}} \right\} \hfill \\ \end{gathered} \right.$$

where ***U*** is the row set of ***P***, $p_{{u{\kern 1pt} v}}^{C}$ refers to the *u*-th site under *v*-th condition in ***P***^***C***^, $e_{{u{\kern 1pt} V^{\prime}}}^{R}$ is calculated based on ***P***^***R***^ and ***W*** for row subset selection, but only the sites involved in ***V′*** are considered. Higher *T*_*R*_ results in less sites in LFB.

Since ***P*** is $p \gg n$, it is intuitive that LFBs will cover more sites than conditions. Thus, |***U′| ≫ ***|***V′|***. The selection of sites needs to be more strict, thus *T*_*R*_ is recommended to be larger for less sites inclusion for each LFB. On the contrary, there are not that much conditions in ***P***, thus, *T*_*C*_ is recommended to be smaller for loose constrain of conditions in each LFB.

In the parameter selection procedure, it is recommended that the upper bound of *T*_*R*_ and *T*_*C*_ be set larger values, and the algorithm can automatically shrink the thresholds range. Although the larger upper bound may introduce computation load, it is still acceptable since no LFBs can be achieved under large thresholds setting. The optimization of *T*_*R*_ and *T*_*C*_ is reached by grid search. With optimized *T*_*R*_ and *T*_*C*_, a subset of sites is randomly selected as ***U′***, and then the subset of conditions ***V′ ***is selected according to (14). ***U′*** and ***V′ ***are updated iteratively by (14) and (15) until convergenece is satisfied.

16 $$\frac{{|\user2{U^{\prime}} \cap \user2{U^{\prime\prime}}|}}{{|\user2{U^{\prime}} \cup \user2{U^{\prime\prime}}|}} \le \varepsilon$$

where *ε* is the default convergence criteria, ***U′′*** represents the subset of sites in previous iteration, and ***U′*** represents the subset of site in current iteration.

The implementation of REW-ISA following the above definition is summarized in the following.

**Table:** 

**Algorithm 2:** REW-ISA biclustering algorithm
**Input:** Methylation site ***V***, conditions ***U***, methylation level matrix ***P***, weight matrix ***W*** and converge threshold *ε*
**Output:** A series of LFBs (***U′***, ***V′***)
**Step1:** Construct row normalized matrix ***P***^***R***^, construct column normalized matrix ***P***^***C***^
**Step2:** Given the pre-defined range for *T*_*R*_ and *T*_*C*_, get the automatically optimized parameter settings
**Step3:** Under the optimized parameters *T*_*R*_ and *T*_*C*_, initialize the sites subset ***U′*** and update ***U′*** and ***V′*** iteratively until the convergence condition is met
**Step4:** Report ***U′*** and ***V′***
**Return** A series of LFBs (***U′***, ***V′***)

## Supplementary information

**Additional file 1. Table S1:** The detailed information of real data. The information of real data. Experiments with light green background were not included in MeTDBV2.0 yet. Experiment ID: The index of experiments; Cell line: The cell line that MeRIP-Seq profiled. Expr_name in MeTDB V2.0: The retrieval information in MeTDBV2.0. If the data was not included in MeTDBV2.0, the GEO accession numbers were provided instead (indicated as light green background). Treatment: The treatment applied for the experiment. Reference: The title of the source reference. Reference ID: Reference number in the article.

## Data Availability

The code implemented to perform the analysis is deposited at https://github.com/cst-cumt/REW-ISA. The detailed information of real data consists of MeRIP-Seq data from 10 cell lines under 32 kinds of treatments, as listed in Additional file 1: Table S1. For each type of treatment, MeRIP-Seq gets input and IP sequences respectively. Among the 32 conditions, 30 can be retrieved from MeTDBV2.0 at https://180.208.58.19/metdb_v2, and the other two can be retrieved by GEO accession numbers (SRR5080301-SRR50312 and SRR5239086-SRR5239109).

## Abbreviations

m^6^A: N^6^-mthyladenosine
REW-ISA: RNA Expression Weighted Iterative Signature Algorithm
LFB: Local functional block
MeRIP-Seq: Methylated RNA immunoprecipitation sequencing
DPBBM: Dirichlet process based beta binomial mixture model
ISA: Iterative signature algorithm
SDwC: Standard deviation within clusters
ASwC: Average similarity within clusters
SoBC: Score of bi-clustering
GO: Gene ontology
IoU: Intersection over union
FPKM: Fragments Per Kilobase of transcript per Million
HGF: Hepatocyte growth factor
HepG2: Human liver hepatocellular cells
